# Pathways and Referral of Patients with Cancer in Rural Ethiopia: A Multi-center Retrospective Cohort Study

**DOI:** 10.1093/oncolo/oyad032

**Published:** 2023-03-20

**Authors:** Josephin Trabitzsch, Abigiya Wondimagegnehu, Tsion Afework, Ole Stoeter, Muluken Gizaw, Sefonias Getachew, Jilcha Diribi Feyisa, Lesley Taylor, Andreas Wienke, Adamu Addissie, Eva Johanna Kantelhardt

**Affiliations:** Global Health Working Group, Martin-Luther-University Halle-Wittenberg, Halle (Saale), Germany; Institute of Medical Epidemiology, Biometrics, and Informatics, Martin-Luther-University Halle-Wittenberg, Halle (Saale), Germany; Global Health Working Group, Martin-Luther-University Halle-Wittenberg, Halle (Saale), Germany; Institute of Medical Epidemiology, Biometrics, and Informatics, Martin-Luther-University Halle-Wittenberg, Halle (Saale), Germany; Department of Preventive Medicine, School of Public Health, Addis Ababa University, Addis Ababa, Ethiopia; Global Health Working Group, Martin-Luther-University Halle-Wittenberg, Halle (Saale), Germany; Department of Preventive Medicine, School of Public Health, Addis Ababa University, Addis Ababa, Ethiopia; Global Health Working Group, Martin-Luther-University Halle-Wittenberg, Halle (Saale), Germany; Institute of Medical Epidemiology, Biometrics, and Informatics, Martin-Luther-University Halle-Wittenberg, Halle (Saale), Germany; Global Health Working Group, Martin-Luther-University Halle-Wittenberg, Halle (Saale), Germany; Institute of Medical Epidemiology, Biometrics, and Informatics, Martin-Luther-University Halle-Wittenberg, Halle (Saale), Germany; Department of Preventive Medicine, School of Public Health, Addis Ababa University, Addis Ababa, Ethiopia; Global Health Working Group, Martin-Luther-University Halle-Wittenberg, Halle (Saale), Germany; Institute of Medical Epidemiology, Biometrics, and Informatics, Martin-Luther-University Halle-Wittenberg, Halle (Saale), Germany; Department of Preventive Medicine, School of Public Health, Addis Ababa University, Addis Ababa, Ethiopia; Global Health Working Group, Martin-Luther-University Halle-Wittenberg, Halle (Saale), Germany; Department of Oncology, Saint Paul’s Hospital Millennium Medical College, Addis Ababa, Ethiopia; Department of Surgery, City of Hope National Medical Center, Duarte, Los Angeles, CA, USA; Institute of Medical Epidemiology, Biometrics, and Informatics, Martin-Luther-University Halle-Wittenberg, Halle (Saale), Germany; Global Health Working Group, Martin-Luther-University Halle-Wittenberg, Halle (Saale), Germany; Institute of Medical Epidemiology, Biometrics, and Informatics, Martin-Luther-University Halle-Wittenberg, Halle (Saale), Germany; Department of Preventive Medicine, School of Public Health, Addis Ababa University, Addis Ababa, Ethiopia; Global Health Working Group, Martin-Luther-University Halle-Wittenberg, Halle (Saale), Germany; Institute of Medical Epidemiology, Biometrics, and Informatics, Martin-Luther-University Halle-Wittenberg, Halle (Saale), Germany; Department of Preventive Medicine, School of Public Health, Addis Ababa University, Addis Ababa, Ethiopia; Department of Gynaecology, Martin-Luther-University Halle-Wittenberg, Halle (Saale), Germany

**Keywords:** cancer, health system, Sub-Saharan Africa, patient pathways

## Abstract

**Introduction:**

Well-organized patient pathways are essential to achieve early diagnosis and timely treatment of patients with cancer in Sub-Saharan Africa. This retrospective cohort study describes pathways and referral patterns of cancer patients in rural Ethiopia.

**Patients and Methods:**

The retrospective study took place from October to December 2020 at 2 primary- and 6 secondary-level hospitals in southwestern Ethiopia. Of 681 eligible patients diagnosed with cancer between July 2017 and June 2020, 365 patients were included. Structured interviews on the patients’ pathways were conducted by telephone. The primary outcome was successful referral, which was defined as occurring when the intended procedure was initiated at the receiving institution. Logistic regression was used to assess factors associated with successful referrals.

**Results:**

Patients visited on average 3 health care institutions from their first encounter with a provider until their final treatment initiation. After diagnosis, only 26% (95) of patients were referred for further cancer treatment, of which 73% were successful. Patients referred for diagnostic tests were 10 times more likely to complete referrals successfully than patients referred for treatment. Overall, 21% of all patients remained without any therapy.

**Conclusion:**

We found that referral pathways of patients with cancer in rural Ethiopia were largely cohesive. The majority of patients referred for diagnostic or treatment services followed the advice. Nevertheless, an unacceptable number of patients remained without any treatment. Capacity for cancer diagnosis and treatment at primary- and secondary-level health facilities in rural Ethiopia must be expanded to enable early detection and timely care.

## Teaser

Disorganized patient referral pathways may cause delays in cancer diagnosis and treatment. This article describes referral patterns and pathways of patients diagnosed at primary and secondary hospitals in rural Ethiopia and assesses factors that contributed to the successful completion of referrals. Opportunities to increase early cancer diagnosis and treatment are identified.

**Implications for Practice**

This study on patient pathways highlights the experiences of patients with cancer diagnosed at primary- and secondary-level hospitals in rural Ethiopia. It includes patients who never reach specialized tertiary care and are therefore missed in studies at specialized cancer centers. An average of 3 care-nodes during the patients’ journey is encouraging. Expanding public pathology services and treatment capacity remain fields of action to enable early cancer diagnosis and treatment for the rural population of Ethiopia.

## Introduction

Advanced stages at diagnosis and long intervals between first symptoms and treatment initiation are causing cancer mortality rates in Sub-Saharan Africa to rank among the highest worldwide.^1^ Disorganized patient pathways have been thought to prevent patients from receiving early diagnosis and treatment.^2,3^ As specialized diagnostic and treatment options are only available in few facilities in Sub-Saharan African countries, efficient referral systems for patients with cancer are of particular importance.

Recent reports on pathways of patients with cancer in Sub-Saharan Africa have largely focused on breast and cervical cancer.^2^ The experiences of patients with less common cancers are not well described. In addition, most data have been collected from tertiary hospitals.^4,5^ Those studies do not describe the unknown proportion of patients who are diagnosed with cancer in peripheral hospitals but never appear in tertiary hospitals to receive treatment. Moreover, there are few detailed studies on referral pathways for patients with suspected cancer.

In Ethiopia, as in many other countries in Sub-Saharan Africa, the health care system is divided into 3 tiers or levels. At the primary level are health posts, health centers, and primary hospitals. At the secondary level are general hospitals, of which larger ones can be affiliated with universities and serve as regional referral hospitals or “secondary referral hospitals.” At the tertiary level are specialized referral hospitals.^6^ In terms of cancer care, primary- and secondary-level hospitals have capacity for diagnosis but limited capacity for general surgical and systemic oncology services. Specifically for breast cancer treatment, endocrine therapy is available at some. For further diagnostics or treatment, such as chemotherapy, patients are mostly referred to one of 7 specialized tertiary referral hospitals around the country, which serve as comprehensive cancer centers. All 7 centers are now equipped with radiation machines and will soon offer treatment to patients. At the time of data collection, Tikur Anbessa Specialized Hospital, located in the capital city Addis Ababa was the only hospital in the country providing radiotherapy.

In this study, we aimed to describe pathways and referral patterns of patients with cancer diagnosed at primary and secondary hospitals in southwestern rural Ethiopia. In addition, we assessed which factors contributed to patients successfully completing referrals. We identify opportunities for future work to increase early cancer diagnosis and treatment.

## Patients and Methods

### Study Design, Setting, and Population

This retrospective cohort study was conducted between October and December 2020 at 8 hospitals in the rural Southwest of Ethiopia (Supplementary Table S1). Two hospitals were primary hospitals, while 3 hospitals were general and secondary referral hospitals. In all hospitals, cancer diagnosis mostly relied on clinical findings or biopsies evaluated at private pathology facilities or specialized hospitals. In terms of treatment, all the hospitals provided basic surgery for common cancers. Endocrine treatment (Tamoxifen) for breast cancer was available at all 8 sites, while only 1 secondary-level referral hospital (Assela University Teaching Hospital) provided chemotherapy.

We compared patient experiences across the primary- and secondary-level of the health-care system. Due to their size and smaller catchment area primary and general hospitals see substantially less patients with cancer compared with secondary referral hospitals. To achieve equal numbers of patients across the 3 groups, the study population consisted of patients aged 18 years or older who were diagnosed with cancer between July 2017 and June 2020 at primary and general hospitals, or between January 2018 and December 2019 for those diagnosed at secondary referral hospitals.

The primary outcome measured in this study was the successful completion of referring patients with cancer for further diagnostics or care. Referrals were defined to be successful when a patient initiated the diagnostic or treatment procedure at the receiving institution. When these procedures were still planned, or could not be determined to have been initiated, we defined the outcome of these referrals as “not determinable.”

### Data Collection

To identify eligible patients, nurses trained in data collection conducted a retrospective case note audit at all sites. The nurses were trained and supervised by our principal data collectors, who were masters-level graduates from the Addis Ababa School of Public Health.

Within the preliminary case note audit, we identified 681 patients diagnosed with cancer, of which 65.3% (445) patients had a morphologically verified diagnosis, while 24% (163) patients had been diagnosed clinically only. For 10% (71) the method of diagnosis was not documented within the case notes. Our principal data collectors reached 370 patients or their relatives via phone to conduct a structured interview about the patient’s referral pathway. The questionnaire was adapted from a validated tool on delay in treatment for breast cancer.^7^ It was discussed with an Ethiopian ­senior-oncologist in advance to ensure validity in the Ethiopian setting. After pretesting the tool with 40 patients at Tikur Anbessa University Hospital few changes were made for contextualized understanding of the questions (Supplementary Table S2). As suggested by Unger-Saldana et al. an algorithm was adapted to the Ethiopian calendar and applied to determine past dates using the calendar technique. If patients were too ill to participate personally or deceased, close relatives were interviewed on their behalf.

Socio-demographic data and clinical data from the study site were cross-checked between patients charts and interviews: For socio-demographic information, the interview data were preferred, and for the patients’ clinical history data from case notes were preferred for accuracy. Of the 370 patients or patients’ relatives interviewed, we excluded 5 patients from further analysis due to incohesive data.

### Patient Pathway Definitions and Referral Processes

We defined patient pathways to comprise broadly of all health care providers the patients saw between their first recognition of cancer symptoms until the completion of their treatment: these included both traditional or spiritual healers and formal health care providers in private or government health care institutions. When a patient accessed a healthcare institution in their pathway, we defined this encounter as a “care node.” When the patient received a diagnosis at a hospital, we defined the location a “study site.” When a patient was referred to another hospital for further diagnostic tests or treatment, we defined that location as the “receiving institution.”

We categorized the reasons for patient referral into 3 groups—for diagnostic tests only, for treatment, or unknown. Patients referred for treatment might also have received diagnostic tests at the receiving institution, however, we assumed treatment to have been the primary objective of the referral. Treatments included surgery, endocrine treatment, chemotherapy, radiation, and radiochemotherapy.

### Data Analysis

Data were entered into EpiData Version 4.6.0.2 and transferred to R Version 4.0.4 for statistical analysis. Descriptive analyses were applied to assess referral patterns as well as diagnostic and treatment initiation intervals.

The influence of predictors on the success of referral was assessed using a logistic regression model. As some patients were referred multiple times, we assessed each referral individually. This way we could include patients who had experienced successful as well as unsuccessful referrals. We used multi-level regression models to investigate cluster effects due to several referrals of one patient, as well as several patients coming from one site. Models were compared using Akaike and Bayesian Information Criteria (AIC and BIC). Both multi-level regression models did not improve the model fit and were therefore discarded.

Predictors were chosen based on literature.^8-10^ Their influence was first checked in univariable regression models. Sex was excluded from the multivariable model, as it was closely correlated with cancer entity and therefore judged to be colinear. Results from the univariable regression are presented as crude odds ratios (COR), those from the multivariable model as adjusted odds ratios (AOR)—both with 95% CIs. Cases where the success of referral was not determinable were excluded from this analysis. However, as they still added valuable data to the descriptive parts of the analysis, we did not remove them from analysis completely.

### Ethical Considerations

This study is part of a project aiming to design, implement, and evaluate decentralized cancer care in Ethiopia. It was approved by the Institutional Review Board of the Addis Ababa University College of Health Science (ref: 041/20/SPH). An ethical protocol regarding interviews with critically ill patients or relatives of deceased patients was implemented and discussed in detail with all members of the data collection team. Study participants gave their oral informed consent before each interview and were offered the investigators´ contact details. All data was handled confidentially and participants´ data were pseudonymized after the phone-calls.

## Results

Of the 365 patients included in the study, 58% answered the phone-call interviews themselves, while 42% of the interviews were conducted with relatives. The main reason why patients did not take part in the interviews themselves, was their death prior to our study: at the time of data collection 31% (113) patients were already deceased. Other reasons for relatives to answer the interview included language barriers as well as the patients’ weak conditions. Patients were predominantly female and breast and cervical cancer made up almost two-thirds of all cancer entities (Table 1). The prevalence of other cancer entities differed strongly between hospitals. Of the total 20 patients with stomach cancer, 90% came from Dubbo St. Mary’s Catholic Hospital (a primary-level hospital site). Also, of the 10 patients with prostate cancer, 50% were diagnosed at Butajira Hospital (a secondary-level general hospital site). Only one hematologic cancer was registered in the cohort.

**Table 1.** Socio-demographic and clinical characteristics of study participants at time of data collection by health-care level of study site.

Variable	All sites (%^a^) *n* = 365	Primary hospital sites (%) *n* = 116	General hospitals sites (%) *n* = 95	Referral hospital sites^b^ (%) *n* = 154
Age (in years, median (IQR))	40 (15)	40 (15)	44.3 (15)	40 (18.8)
Sex				
Female	283 (77.5)	76 (65.5)	81 (85.3)	126 (81.8)
Male	82 (22.5)	40 (34.5)	14 (14.7)	28 (18.2)
Religion				
Orthodox	155 (42.5)	51 (44)	42 (44.2)	62 (40.3)
Protestant	125 (34.2)	46 (39.7)	25 (26.3)	54 (35.1)
Muslim	80 (21.9)	18 (15.5)	25 (26.3)	37 (24)
Other^c^	5 (1.4)	1 (0.9)	3 (3.2)	1 (0.6)
Educational level				
No formal education	187 (51.2)	80 (69)	39 (41.1)	68 (44.2)
Primary	107 (29.3)	26 (22.4)	42 (44.2)	39 (25.3)
Secondary or above	65 (17.8)	9 (7.8)	14 (14.7)	42 (27.3)
Unknown	6 (1.6)	1 (0.9)	0 (0)	5 (3.2)
Occupation				
Housewife	216 (59.2)	64 (55.2)	64 (67.4)	88 (57.1)
Farmer	82 (22.5)	38 (32.8)	20 (21.1)	24 (15.6)
Civil servant	32 (8.8)	6 (5.2)	8 (8.4)	18 (11.7)
Other^d^	33 (9)	8 (6.9)	2 (2.1)	23 (14.9)
Unknown	2 (0.5)	0 (0)	1 (1.1)	1 (0.6)
Marital status				
Married	312 (85.5)	110 (94.8)	73 (76.8)	129 (83.8)
Single	26 (7.1)	5 (4.3)	4 (4.2)	17 (11)
Widowed	20 (5.5)	0 (0)	13 (13.7)	7 (4.5)
Divorced/separated	7 (1.9)	1 (0.9)	5 (5.3)	1 (0.6)
Cancer entity				
Breast	166 (45.5)	44 (37.9)	45 (47.4)	77 (50)
Cervix	65 (17.8)	9 (7.8)	30 (31.6)	26 (16.9)
Colorectum	32 (8.8)	13 (11.2)	4 (4.2)	15 (9.7)
Stomach	20 (5.5)	18 (15.5)	2 (2.1)	0 (0)
Other^e^	82 (22.5)	32 (27.6)	14 (14.7)	36 (23.4)
Stage				
I	10 (2.7)	2 (1.7)	3 (3.2)	5 (3.2)
II	44 (12.1)	10 (8.6)	14 (14.7)	20 (13)
III	99 (27.1)	15 (12.9)	47 (49.5)	37 (24)
IV	49 (13.4)	7 (6)	17 (17.9)	25 (16.2)
Not documented	163 (44.7)	82 (70.7)	14 (14.7)	67 (43.5)
Method of diagnosis as documented on site				
Clinically	78 (21.4)	49 (42.2)	11 (11.6)	18 (11.7)
Morphologically verified	252 (69)	60 (51.7)	73 (76.8)	119 (77.3)
Not documented	35 (9.6)	7 (6)	11 (11.6)	17 (11)
Place of morphological verification^f^				
Study site	74 (29.4)	4 (6.7)	36 (49.3)	34 (28.6)
Private sector	159 (63.1)	54 (90)	32 (43.8)	73 (61.3)
Other^g^	18 (7.1)	2 (3.3)	4 (5.5)	12 (10.1)
Unknown	1 (0.4)	0 (0)	1 (1.4)	0 (0)
Therapy on study site^h^				
Surgery suggested	203 (55.6)	50 (43.1)	72 (75.8)	81 (52.6)
Surgery performed	180 (88.7)	42 (84)	65 (90.3)	73 (90.1)
Endocrine treatment suggested	101 (27.7)	38 (32.8)	39 (41.1)	24 (15.6)
Endocrine treatment initiated	98 (97)	37 (97.4)	37 (94.9)	24 (100)
Chemotherapy suggested	53 (14.5)	0 (0)	0 (0)	53 (34.4)
Chemotherapy initiated	53 (100)	0 (0)	0 (0)	53 (100)
Therapeutic intent				
Curative	102 (27.9)	9 (7.8)	50 (52.6)	43 (27.9)
Palliative	110 (30.1)	30 (25.9)	23 (24.2)	57 (37)
Not determinable^i^	153 (41.9)	77 (66.4)	22 (23.2)	54 (35.1)
No. of patients deceased	113 (31)	55 (47.4)	29 (30.5)	29 (18.8)

^a^Column wise percentage unless otherwise specified.

^b^Referral hospitals on the secondary-level of the health-care level.

^c^Other religions include catholic religion and atheists.

^d^Other occupations include student, teacher, factory worker, merchant, day labourer, machine operator.

^e^Other cancer entities include prostate cancer, esophageal cancer, thyroid cancer, ovarian cancer, liver cancer, bladder cancer and others.

^f^Percentages are in relation to number of patients with morphologically verified diagnosis.

^g^Other place of morphological verification includes primary, secondary, and tertiary health care facilities.

^h^Percentages for performed/initiated procedures are in relation to number of suggested procedures.

^i^If therapeutic intent was not documented, stage I was estimated curative, stage IV palliative, stages II and III were rated not determinable.

A morphologically verified diagnosis was available in the medical records of 69% (252) patients. At primary-level hospitals, most pathology review was performed in the private sector. Cancer stage at time of diagnosis was documented for 55.3% (202) patients. Almost 3 quarters of those patients had been diagnosed with cancer stages III or IV.

At hospitals where patients were diagnosed with cancer (the study sites), 49% (180) of patients received surgery, 27% (98) endocrine treatment, and 14% (53) chemotherapy. When doctors at study sites made treatment recommendations, the majority of patients accepted endocrine and chemotherapy, and 89% of patients accepted surgery.

### Patient Pathways

Table 2 shows that 220 (60%) patients went to a primary level health center when first seeking help for their cancer symptoms. On average, patients visited 1.2 health-care facilities before their diagnosis at the study site. The average number of care nodes visited throughout the entire patient pathway (from first seeking medical advice until final treatment initiation) was 3. Only 35 (10%) patients reported visiting a traditional or spiritual healer after their first cancer symptom recognition.

**Table 2.** Characteristics of patients’ with cancer pathways by health-care level of study site.

	All (%^a^) *n* = 365	Primary hospital sites (%) *n* = 115	General hospital sites (%) *n* = 95	Referral^b^ hospital sites (%) *n* = 154
First health care provider visited with cancer symptoms				
Traditional or spiritual healer	31 (8.5)	6 (5.2)	2 (2.1)	23 (14.9)
Health post	21 (5.8)	4 (3.4)	4 (4.2)	13 (8.4)
Health center	220 (60.3)	77 (66.4)	53 (55.8)	90 (58.4)
Primary hospital	60 (16.4)	23 (19.8)	20 (21.1)	17 (11)
Other^c^	19 (5.2)	3 (2.6)	9 (9.5)	7 (4.5)
Unknown	14 (3.8)	3 (2.6)	7 (7.4)	4 (2.6)
No. of care nodes visited^d,e^ (mean (SD))				
Before diagnosis at study site	1.2 (0.66)	1 (0.76)	1 (0.49)	1.4 (0.58)
After diagnosis at study site	0.8 (0.68)	1.2 (0.73)	0.8 (0.51)	0.5 (0.52)
During total pathway	3 (0.85)	3.2 (0.97)	2.8 (0.76)	2.9 (0.76)
Distribution of total care nodes				
1–2 care nodes	89 (24.4)	24 (20.7)	22 (23.2)	43 (27.9)
3–4 care nodes	230 (63)	77 (66.4)	65 (68.4)	88 (57.1)
5–6 care nodes	12 (3.3)	10 (8.6)	0 (0)	2 (1.3)
Pathway not finished	13 (3.6)	2 (1.7)	3 (3.2)	8 (5.2)
Unknown	21 (5.8)	3 (2.6)	5 (5.3)	13 (8.4)
Patients ever visiting a traditional or spiritual healer since first symptom recognition	35 (9.6)	13 (3.6)	2 (0.5)	20 (5.5)

^a^Column wise percentage unless otherwise specified.

^b^Referral hospitals on the secondary-level of the health-care level.

^c^Other includes secondary-level hospitals or private health care facilities.

^d^Care nodes are defined as formal health care facilities patients addressed on their pathway.

^e^Patients whose pathway was not known to be finished are excluded (*n* = 13).

### Referral Patterns

After patients were diagnosed with cancer at study sites, 250 (68%) were referred once to another facility, and 50 (14%) were referred twice (Table 3). At both primary level and general hospital study sites, first referrals were mostly for diagnostic tests only, and second referral were for treatment. At secondary-level referral hospital sites, the reasons for referrals were evenly distributed between diagnostic tests and treatment. A second referral hardly ever occurred. All in all, after diagnosis 26% (95) of patients were referred for any further cancer treatment.

**Table 3.** Primary objectives and success of referrals from study site by health-care level of study site.

	All sites (%) *n* = 365	Primary hospital sites (%) *n* = 116	General hospital sites (%) *n* = 95	Referral^a^ hospital sites (%) *n* = 154
Primary objectives of referral (% of all first or second referrals)				
First referral (*n* = 250)				
Diagnostics only	167 (66.8)	87 (87)	44 (60.3)	36 (46.8)
Treatment	72 (28.8)	8 (8)	28 (38.4)	36 (46.8)
Objective unknown	11 (4.4)	5 (5)	1 (1.4)	5 (6.5)
Second referral^b^ (*n* = 50)				
Treatment	27 (54)	20 (47.6)	5 (83.3)	2 (100)
Objective unknown	23 (46)	22 (52.4)	1 (16.7)	0 (0)
Referrals’' success by primary objective (% of all referrals for given objective)				
Diagnostics only (*n* =167)				
Successful	161 (96.4)	86 (98.9)	42 (95.5)	33 (91.7)
Not successful	4 (2.4)	1 (1.1)	1 (2.3)	2 (5.6)
Success not determinable ^c^	2 (1.2)	0 (0)	1 (2.3)	1 (2.8)
Treatment (*n* = 99)				
Successful	72 (72.7)	21 (75)	26 (78.8)	25 (65.8)
Not successful	19 (19.2)	7 (25)	6 (18.2)	6 (15.8)
Success not determinable	8 (8.1)	0 (0)	1 (3)	7 (18.4)
Objective unknown^d^ (*n* = 34)				
Not successful	32 (94.1)	25 (92.6)	2 (100)	5 (100)
Success not determinable	2 (5.9)	2 (7.4)	0 (0)	0 (0)

^a^Referral hospitals on the secondary-level of the health-care level.

^b^No referrals for diagnostics only among second referrals.

^c^Success of referral was classified “not determinable” if treatment initiation was pending or unknown at time of data collection. The number of patients referred for diagnostics only with pending referral status was 0, the number of patients referred for treatment with pending referral status was 8. There were no pending referrals among patients with unknown referral objective.

^d^No successful referrals among referrals with unknown objective.

Success rates of referrals differed strongly between the reason for the referral. The referral was more successfully completed for diagnostic tests than for initiation of treatment. While 96% (161) of the referrals for diagnostic procedures were successful, only 73% (72) of referrals for treatment resulted in treatment initiation at the receiving institutions.

**Figure 1.**
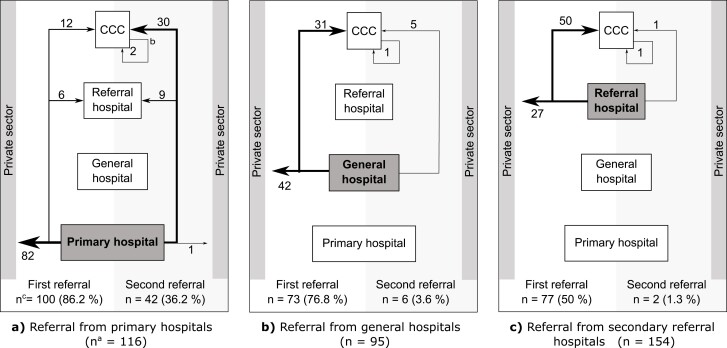
Referral pathways of patients with cancer along the 3-tier health care system in Ethiopia. Patients were diagnosed (and included into the study) at (**a**) primary hospital, (**b**) general hospital, or (**c**) secondary referral hospital level and then referred for diagnostic tests or treatment. CCC, comprehensive cancer center (tertiary specialized hospital). ^**a**^*n* corresponds to total number of patients in study cohort registered on specific health care level. ^**b**^Arrow corresponds to referrals within health-care level. ^**c**^*n* corresponds to number of patients referred of all patients in study cohort registered on specific health-care level.

Figure 1 shows the patient experience of referrals after receiving a cancer diagnosis at study sites. At primary-level hospital study sites, patients were first referred to the private sector usually for additional diagnostic services. Often a second referral was then made to receive care at a comprehensive cancer center. By contrast, at secondary-level hospitals, 65% (50) of patients were referred directly to a comprehensive cancer center. Only one primary hospital site referred patients to a secondary referral hospital.

### Perceived Challenges and Enablers to Completing a Referral

Patients identified two major challenges to completing a referral: 73% (268) reported financial hardship in affording diagnostic tests and treatment; 30% (110) reported transportation issues. While 11% (41) described a lack of social support, most patients (76%, 279) stated that family support enabled them to complete their referrals. Perceptions of challenges and enablers did not substantially differ between patients who had been referred successfully and patients whose referral had not been successful.

### Predictors of Successful Referral

Patients were 10 times more likely to complete the referral successfully when the reason was for diagnostic tests rather than treatment (Table 4). The trend increased in the multivariable model to an odds ratio of 13.3 (CI 4.12–42.92). In univariable regression, the other factors associated with successful referrals were being female, referral from a general hospital, and being diagnosed with breast or cervical cancer. These factors were not confirmed in the multivariable model. The remaining tested predictors (age, religion, occupation, education, and stage) did not show any association with successful referral.

**Table 4.** Crude odds ratio (COR) and adjusted^a^ odds ratio (AOR) with 95% CIs for successful referral in study cohort.

	Referrals^b^ *N* = 288	Successful referrals (%)	COR (CI)	*P*-value^c^	AOR (CI)	*P*-value^d^
Sex						
Male	71	51 (71.8)	Reference	—	—	—
Female	217	182 (83.9)	2.04 (1.08, 3.83)	.03	—	—
Age (in years)						
>40	134	102 (76.1)	Reference	—	—	—
≤40	154	131 (85.1)	1.79 (0.99, 3.24)	.06	1.74 (0.68, 4.44)	.25
Religion						
Protestant	106	83 (78.3)	Reference	—	—	—
Orthodox	129	102 (79.1)	1.05 (0.56, 1.96)	.89	—	—
Other^e^	53	48 (90.6)	2.66 (0.95, 7.45)	.06	—	—
Occupation						
Farmer	65	47 (72.3)	Reference	—	—	—
Housewife	170	141 (82.9)	1.86 (0.95, 3.66)	.07	—	—
Civil servant	29	24 (82.8)	1.84 (0.61, 5.56)	.28	—	—
Other^f^	24	21 (87.5)	2.68 (0.71, 10.1)	.14	—	—
Marital status						
Not married	35	30 (85.7)	Reference	—	—	—
Married	253	203 (80.2)	0.68 (0.25, 1.83)	.44	—	—
Education						
No formal education	152	120 (79)	Reference	—	—	—
Primary	86	71 (82.6)	1.26 (0.64, 2.49)	.5	—	—
Secondary or higher	50	42 (84)	1.4 (0.6, 3.28)	.44	—	—
Health care level of study site						
Primary hospital	140	107 (76.4)	Reference	—	—	—
General hospital	77	68 (88.3)	2.33 (1.05, 5.17)	.04	0.76 (0.22, 2.63)	.67
Secondary referral hospital	71	58 (81.7)	1.38 (0.67, 2.82)	.38	0.71 (0.22, 2.26)	.56
Cancer entity	—	—	—	—	—	—
Other^g^	119	84 (70.6)	Reference	—	—	—
Breast	108	97 (89.8)	3.67 (1.76, 7.68)	<.01	1.38 (0.44, 4.33)	.58
Cervix	61	52 (85.2)	2.41 (1.07, 5.41)	.03	2.92 (0.83, 10.33)	.1
Stage	—	—	—	—	—	—
I—II	39	34 (87.2)	Reference	—	—	—
III—IV	96	86 (89.6)	1.26 (0.4, 3.97)	.69	—	—
Unknown	153	113 (73.9)	0.42 (0.15, 1.14)	.09	—	—
Primary objective of referral						
Treatment	91	72 (79.1)	Reference	—	—	—
Diagnostics only	165	161 (97.6)	10.62 (3.5, 32.34)	<.01	13.3 (4.12, 42.92)	<.01
Unknown^h^	32	0 (0)	—	—	—	—

^a^Adjusted for age, health-care level of study site, cancer entity and objective of referral.

^b^Referrals where procedures were still planned or not known to have been initiated were excluded (*n* = 12).

^c^ *P*-value for crude odds ratio.

^d^ *P*-value for adjusted odds ratio.

^e^Other religions include catholic religion and atheists.

^f^Other occupations include student, teacher, factory worker, merchant, day labourer, machine operator.

^g^Other cancer entities includes prostate cancer, oesophageal cancer, thyroid cancer, ovarian cancer, liver cancer, bladder cancer and others.

^h^Unknown objectives were not included into regression models due to no successful referrals.

### Extent of Overall Cancer Treatment

**Figure 2.**
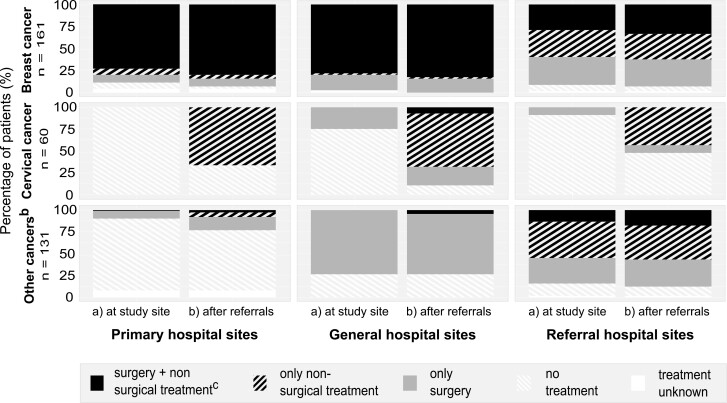
Treatment received by patients^a^ with cancer (**a**) at study site and (**b**) after referrals in Ethiopia. ^**a**^Excluding patients whose pathway was not finished or not known to be finished (*n* = 13). ^**b**^Other cancers include prostate cancer, oesophageal cancer, thyroid cancer, ovarian cancer, liver cancer, bladder cancer, and others. ^**c**^Non-surgical treatment includes hormonal treatment, chemotherapy, radiochemotherapy, and radiotherapy.

Figure 2 shows how the referral patterns impacted the patient’s initiation of treatment.

On the study sites (primary or secondary hospitals) 63% (221) of patients initiated some form of treatment. After referrals, this number increased to 76% (269). We found that 21% of patients remained without any treatment—even after referrals.

Overall, treatment differed between cancer entities and study sites. At primary and secondary general hospitals, 75% (67) of patients with breast cancer received surgery and non-surgical treatment without referral. At secondary-level referral hospitals only 29% (21) received surgery and non-surgical treatment on-site. Across all levels, only 5% of all patients with breast cancer did not receive any therapy at a study site, which was reduced to 3% after referrals.

By contrast, only 15% (9) of all patients with cervical cancer across all study sites received any form of treatment on-site, regardless of where they received their diagnosis. After referrals, 72% (43) of the patients with cervical cancer received some sort of treatment, however, 28% (17) remained untreated.

For other cancers (*n* = 131) only 56% (73) of patients received any form of therapy, even after referral. In these cases, the initiation of treatment strongly depended on the health-care level at where patients received their diagnosis. For patients diagnosed at primary-level hospital sites, 23% (14) of patients initiated any form of treatment after referral. When patients were diagnosed at general and secondary referral hospital study sites, those numbers were higher: 74% (14) and 88% (45), respectively.

## Discussion

In this study, we described pathways and referral patterns of patients diagnosed with cancer at primary- and ­secondary-level hospitals in the rural southwestern region of Ethiopia. We found fewer than one-third of patients were referred for treatment from the study sites—however, those patients who were referred largely followed the referral advice. One-fifth of all patients remained without any cancer treatment.

As the Ethiopian government has been investing in developing the primary health care level for the last 2 decades, it was satisfactory to find referral pathways at this level are working as intended.^11^ More than 80% of patients in our cohort accessed the health-care system at the primary level. After having addressed a health post or health center with their cancer symptoms, most patients were referred directly to a primary- or secondary-level hospital. The average number of care nodes visited before diagnosis at study sites ranged between 1 when patients were seen at primary and general hospitals, and 1.4 at secondary referral hospitals. The observation that 60% of all patients first sought assessment for cancer symptoms at a health center highlights the importance of primary level facilities in early detection of cancer.

In contrast to previous claims about disorganized referral pathways, we found cohesive referral patterns after patients received clinical or pathological diagnosis at the primary- or secondary-level health care sites.^2,5^ Most patients were either referred to the private sector for confirmatory diagnostic tests or to a comprehensive cancer center directly. On average, patients visited 3 facilities (care nodes), consistent with findings from a mixed-method study on cervical cancer patients conducted at Tikur Anbessa Specialized Hospital in 2013.^12^ This suggests that most patients followed pathways along the 3-tier health care system, largely bypassing multiple referrals within one health care level or counter-referrals.

The strongest predictor for a successful referral in our model was the referral objective. Patients were greater than 10 times more likely to complete the referral successfully when the reason was for diagnostic tests rather than treatment. Diagnostic services for cancer patients in this rural region of Ethiopia are offered by private clinics or hospitals situated in small towns. This keeps additional indirect costs for diagnostic procedures (transport, being away from work, and accommodation) lower than seeking diagnostic services in distant larger cities. Moreover, diagnostic tests are cheaper than total treatment costs. Patients may be more likely to compete for their referral for diagnostic tests than for treatment because of these financial considerations. Although access to diagnostic services is more broadly available than treatment, patients still experience significant delays between their clinical presentation and obtaining a morphological diagnosis.^2^ A study about patients with breast cancer in rural Ethiopia confirmed our finding, that most patients had to be referred for a morphologically verified diagnosis—prolonging time to diagnosis.^13^ Establishing pathology services at primary- and secondary-level hospitals or a well-defined collaboration within the private sector could cut referrals for diagnostic services and therefore shorten time to diagnosis. Implementation of such an intervention would align with the World Health Organization (WHO) Global Breast Cancer Initiative to decrease diagnostic time intervals to <60 days.^14^

Overall, we found higher than expected success rates of patients following through with referrals. Referrals for treatment were successful in 73% of cases. In a WHO trial evaluating referral of women after a positive visual inspection with acetic acids in Tanzania, rates of successful referrals only ranged between 36% and 56%.^15^ Similar to our findings, that study did not show a significant impact of sociodemographic characteristics on the successful completion of referrals.^16^ However, we did not include the impact of the patient´s financial situation in the regression model due to low response rates. Considering almost 3 quarters of all respondents had reported cost barriers associated with the referrals, the influence of the patient’s economic status on successful referrals was notable.

Opportunities exist to increase the rate of referrals, as less than a third of patients reported a referral for treatment after receiving a cancer diagnosis. This might be explained by the patients’ mostly advanced cancer stage at time of diagnosis. However, reasons why health care professionals at primary- and secondary-level hospitals do not refer in the first place remain to be investigated for opportunities in education and areas of improvement.

We found 21% of rural patients with cancer received no treatment even after referrals. This is in line with data from the population-based Addis Ababa Cancer Registry where one-fifth of all patients diagnosed with cancer between 2012 and 2014 remained without any therapy even in the capital city, with presumed access to care.^17^

Patients with malignancies other than cervical or breast cancer seem to face significant hardship when seeking care. Multiple cancers, such as blood cancers were greatly underrepresented within our study cohort compared to relevant incidence rates reported from Addis Ababa.^18^ This suggests that certain patients might not receive a cancer diagnosis in the first place. Of those who were diagnosed, chances of receiving any therapy were low at primary-level hospital study sites, where almost 70% of all patients diagnosed with cancer other than breast or cervical cancer remained untreated. Even though the current focus on breast and cervical cancer seems justified due to their absolute numbers, awareness, and education about other cancer entities at primary and secondary health-care levels must increase urgently.

We consider it a great strength of this study to have assessed pathways of cancer patients registered at the primary and secondary health-care levels, enabling us to describe the experiences of patients who might never have been included in patient cohorts at tertiary hospitals.

The study has several limitations. First is the retrospective, hospital-based sampling method. Only 58% of the patients identified in the chart note audit could be reached via telephone and more than a third of all interviews were conducted with relatives. It is possible patients diagnosed at advanced stages or patients with unsuccessful referrals resulting in less treatment had higher likelihood of severe disease or death before the time of data collection. Such patients were therefore likely not included and underrepresented in this study.

Second, this study may have recall bias. To reach adequate sample size, we had to create a 3-year eligibility period for diagnosis at primary and general hospitals and 2 years eligibility period at secondary referral hospitals. We tried to minimize the resulting recall-bias by using the calendar technique for determination of dates and cross-checking with patient charts wherever possible.

Third, we did not ask patients whether they received any treatments before diagnosis at the study site. Therefore, the described extent of treatment received at secondary-level referral hospitals might be incomplete. This might explain the low numbers of patients with breast cancer at secondary referral hospitals receiving surgery and non-surgical treatments.

Lastly, the number of patients with incomplete pathways in this study (*n* = 13) seems small, considering the long waiting times for treatment at secondary and tertiary referral hospitals.^19^ Some pathways might have ended prematurely due to the patient’s death and should not have been counted as “not successful.” We were unable to inquire the direction of causality between non-referral and death from relatives. Also, we could not entirely rule out the possibility that patients might have received further treatment after the time of data collection, even though no further treatment had been planned at that time.

## Conclusions

Pathways of patients with cancer in rural Ethiopia followed a largely cohesive pattern from the primary and secondary levels to tertiary level care—which points toward considerable awareness among patients and health workers in that region. While the number of patients referred for treatment is low, those patients who are referred mostly follow the referral advice.

It was encouraging to find the majority of patients with breast cancer being offered and accepting surgery and systemic treatment at primary and secondary health care level. Expanding capacity in diagnosis and treatment for other cancer entities could reduce the considerable number of patients still remaining without any cancer treatment. While diagnostic and treatment services are expanding to all health-care levels, barriers preventing health care providers in peripheral hospitals from referring patients for specialized cancer treatment need to be identified and addressed.

## Supplementary Material

oyad032_suppl_Supplementary_Table_S1

oyad032_suppl_Supplementary_Table_S2

## Data Availability

The data underlying this article will be shared on reasonable request to the corresponding author.
