# Supplementary material for: Pathways and Referral of Patients with Cancer in Rural Ethiopia: A Multi-center Retrospective Cohort Study
Source: Oncologist. 2023 Mar 20;28(6):e359–68. doi: 10.1093/oncolo/oyad032 (PMC10243765; doi:10.1093/oncolo/oyad032)
Supplement: oyad032_suppl_Supplementary_Table_S1 [file oyad032_suppl_supplementary_table_s1.docx]

| Supplementary Table **1 \|** Capacity, staff, and equipment available at study sites at time of data collection (December 2020). | | | | | | | | |  |  |
| --- | --- | --- | --- | --- | --- | --- | --- | --- | --- | --- |
|  | **Attat Our First Lady of Lourdes Catholic Primary Hospital** | **Dubbo St. Mary Catholic Hospital** | **St. Lucas Catholic Hospital** | **Butajira General Hospital** | **Dr. Bogalech Memorial Hospital, Durame** | **Assela University Teaching and Referral Hospital** | **Negist Elleni Mohamed Memorial Referral Hospital** | **Wolaita Sodo University Teaching and Referral Hospital** |  |  |
| Hospital level | Primary hospital | Primary hospital | Secondary general hospital | Secondary general hospital | Secondary general hospital | Secondary referral hospital | Secondary referral hospital | Secondary referral hospital |  |  |
| Region and Zone | Gurage Zone,  SNNP | Wolaita Zone,  SNNP | S.-W. Shewa, Oromia | Gurage Zone, SNNP | Kembata Tembaro, SNNP | Arsi Zone, Oromia | Hadiya Zone, SNNP | Wolaita Zone,  SNNP |  |  |
| Catchment population | 800,000 | 800,000 | 1,300,000 | 1,300,000 | 1,000,000 | 3,000,000 | 3,000,000 | 2,000,000 |  |  |
| No. of beds | 65 | 100 | 200 | 190 | 100 | 350 | 220 | 200 |  |  |
| **Staff**: |  |  |  |  |  |  |  |  |  |  |
| No. of doctors | 6 | 6 | 18 | 31 | 25 | 98 | 108 | 134 |  |  |
| No. of oncologists | 0 | 0 | 0 | 0 | 0 | 1 | 0 | 1 |  |  |
| No. of pathologists | 0 | 0 | 0 | 0 | 0 | 2 | 1 | 1 |  |  |
| **Diagnostics available on site:** | | | | | | | | |  | |
| X-ray | yes | no | yes | yes | yes | yes | yes | yes |  |  |
| CT | no | yes | no | no | no | no | no | yes |  |  |
| MRI | no | no | no | no | no | no | no | on installation |  |  |
| FNAC | no | no | yes | no | no | no | yes | yes |  |  |
| Biopsy^a^ | no | no | no | no | no | no | on installation | no |  |  |
| **Treatment available on site**: | | | | | | | | |  | |
| Chemotherapy | no | no | no | no | no | yes | no | no |  |  |
| Hormonal Therapy | Tamoxifen | Tamoxifen | Tamoxifen | Tamoxifen | Tamoxifen | Tamoxifen, Anastrazole | Tamoxifen | Tamoxifen |  |  |
| Pain medication | yes | yes | yes | yes | yes | yes | yes | yes |  |  |
| **Referral:** |  |  |  |  |  |  |  |  |  |  |
| Distance to Addis (km) | 175 | 300 | 130 | 130 | 275 | 165 | 230 | 365 |  |  |
| Referral hub hospital | Alert Hospital/TASH | Wolaita Sodo Hospital | TASH/SPMH | SPMH/  TASH | Hawassa Hospital | TASH | TASH | Hawassa Hospital, TASH |  |  |
| ***SNNP*** Southern Nations, Nationalities, and Peoples; ***TASH*** Tikur Anbessa Specialized Hospital; ***Wolayta Sodo Hospital*** Wolaita Sodo University Teaching and Referral Hospital; ***SPMH*** St.Paul´s Millenium Hospital; ***Hawassa Hospital*** Hawassa University Comprehensive Specialized Hospital  ^a^Biopsies are mostly taken on study sites and sent to private laboratories or tertiary health care facilities for evaluation. | | | | | | | | | |  |
