# Supplementary material for: Pathways and Referral of Patients with Cancer in Rural Ethiopia: A Multi-center Retrospective Cohort Study
Source: Oncologist. 2023 Mar 20;28(6):e359–68. doi: 10.1093/oncolo/oyad032 (PMC10243765; doi:10.1093/oncolo/oyad032)
Supplement: oyad032_suppl_Supplementary_Table_S2 [file oyad032_suppl_supplementary_table_s2.docx]

**Supplementary Table 2 | Questionnaire**

**Telephone Follow-up on Referral Patterns between Health Care Levels**

1. **Introduction:**

| 101 | StudyID  ***To match from data collection tool*** |  | |
| --- | --- | --- | --- |
| 102 | Medical registration number: |  | |
| 103 | Name of the hospital: |  | |
| 104 | Start of interview | *enter time (HH/MM)* | |
| Good morning, my name is (…) and I am a researcher from the School of Public Health in Addis Ababa. We are conducting a study on the experiences of patients who have been diagnosed with illness of the ***(fill in primary cancer site according to case note data***) in a regional hospital in the past two years. Our goal is to use the results of the survey, to provide easier and faster diagnosis and treatment for patients in rural Ethiopia. | | | |
| 105 | Would you have 15 minutes time to answer some questions about your experiences within the health system? | 1. Yes 2. No (continue 107) |  |
| Informed consent | | | |
| You have been randomly selected to participate in this study, and your participation is completely on a voluntary basis. You are free to ask me any questions after the interview or if you need any clarity you may stop me during the interview so that I may assist you. You can also ask to pause, end, or postpone the interview at any time during the interview without consequence. I´ll be happy to leave you a contact number, so you can reach us any time after the interview, if you have any questions or concerns about the survey or your illness.  I´ll take notes during the interview and I will record the call. However, the recordings will be strictly used for this research project and will not be accessible to people outside this research team. I will not note down your name, so any information you provide will be handled anonymously and strictly confidentially. | | | |
| 106 | Is it okay for me to continue with the interview? | 1. Yes (continue 108) 2. No |  |
| 107 | Can I call back another time? | 1. Yes 2. No | If yes: When? |
| 108 | Whom is the interview conducted with? | 1. Patient (continue 201) 2. Spouse of the patient 3. Brother/sister of the patient 4. Child of the patient 5. Other: | If “other”, please specify. |
| **Information about the respondent in case interview is not conducted with patient.**  **If interview is conducted with the patients, please continue at 201.** | | | |
| 109 | Age of the respondent |  | If not answered enter -99 |
| 110 | Relationship of the respondent with the patient: | 1. Lived in the same household as patient 2. Lived in the same village as patient 3. Other 4. Not answered |  |
| 111 | Reason for not conducting the interview with the patient herself/himself | 1. Patient is too sick to talk **(continue 113)** 2. Patient died 3. Other: **(continue 113)** 4. Not answered | If “other”, please specify. |
| 112 | Date of death of patient |  | DD/MM/YYYYY  Use calendar method.  If not answered enter 01/01/1888 |
| 113 | Does the respondent know about the situation and treatment of the patient during his/her illness? | 1. Yes 2. No (stop interview) 3. Not answered |  |

1. **Demographics of the patient**

| 201 | Age |  | If not answered enter -99 |
| --- | --- | --- | --- |
| 202 | Place of residence | Region:  Zone:  Woreda: | If not answered enter “NA”. |
| 203 | Religion | 1. Orthodox 2. Muslim 3. Protestant 4. Catholic 5. Traditional 6. Other: 7. Not answered | If “other”, please ask for specification. |
| 204 | Level of education attended | 1. Illiterate 2. Read and write 3. Primary education (Grade 1-8) 4. Secondary education (Grade 9-12) 5. Diploma 6. Degree and above 7. Not answered |  |
| 205 | Occupation | 1. Housewife 2. Farmer 3. Civil servant in office 4. Factory worker 5. Other: 6. Not answered | If “other”, please ask for specification. |
| 206 | Monthly household income in birr |  | If not answered enter -99 |
| 207 | Marital status | 1. Single 2. Married 3. Divorced 4. Widowed 5. Separated 6. Not answered |  |

1. **Symptoms and Diagnosis**

| 301 | When had you first been having symptoms linked to your disease? |  | MM/YYYYY  Use calendar method.  If not answered enter 01/01/1888 |
| --- | --- | --- | --- |
| 302 | Which was the first health facility you visited when seeking help? | 1. Traditional or spiritual healer 2. Health post 3. Health centre 4. Primary hospital 5. General hospital 6. Other: 7. Not answered | If “other”, please ask for specification. |
| 303 | How many health facilities did you visit before coming to this hospital *(as specified in 103)?* |  | *Traditional or spiritual healers excluded*.  *If not answered enter -99.* |
| 304 | When were you diagnosed? |  | DD/MM/YYYY  Use calendar method.  If not answered enter 01/01/1888 |
| 305 | Where were you diagnosed? | 1. Hospital as specified in 103 2. Other: 3. Not answered | If “other”, please ask for specification. |
| 306 | Which disease were you diagnosed with? | 1. Cancer of the breast 2. Cancer of the cervix 3. Cancer of the colorectum 4. Prostate cancer 5. Oesophageal cancer 6. Other: 7. Not answered | If “other”, please ask for specification. |
| 307 | Where you diagnosed by taking very small tissue (FNAC/ biopsy)? | 1. Yes 2. No (continue 401) 3. Don´t know (continue 401) 4. Not answered (continue 401) |  |

1. **Original therapy at surveyed hospital (as specified in Q103)**

| 401 | Did you receive surgery at hospital as specified in 103? | 1. Yes 2. No (continue 404) 3. Surgery is planned 4. Not answered (continue 404) | Choose “planned” if appointment is made. |
| --- | --- | --- | --- |
| 402 | Which surgery did you receive at the hospital/ will you be receiving at the regional hospital? | 1. Breast surgery (mastectomy) 2. Removal of the uterus (hysterectomy) 3. Colon surgery (colostomy) 4. Removal of the prostate (prostatectomy) 5. Removal of the orchids (orchidectomy) 6. Other: 7. Don´t know 8. Not answered | If “other”, please ask for specification. |
| 403 | When did you receive surgery/ will you be receiving surgery? |  | DD/MM/YYYY  Use calendar method.  If not answered enter 01/01/1888 |
| 404 | Did you receive hormonal treatment at the hospital? | 1. Yes 2. No (continue 406) 3. Planned, but not yet initiated 4. Not answered (406) | ***Probe:***  *Did you get drugs, which you would have to take daily?* |
| 405 | When did you start receiving hormonal treatment? |  | *Date of initiation*  *DD/MM/YYYY*  Use calendar method.  If not answered enter 01/01/1888 |
| 406 | Did you receive chemotherapy at the hospital? | 1. Yes 2. No (continue 408) 3. Planned, but not yet initiated 4. Not answered (continue 408) | ***Probe:***  *Did you receive regular infusions at hospital?* |
| 407 | When did you start receiving chemotherapy? |  | *Date of initiation*  *DD/MM/YYYY*  Use calendar method.  If not answered enter 01/01/1888 |
| 408 | Did you receive any continuous pain medication? | 1. Yes 2. No (continue 501) 3. Not answered (continue 501) |  |

1. **Referral**

| 501 | Were you referred to another health facility? | 1. Yes 2. No (continue 701) 3. Not answered (continue 701) |  |
| --- | --- | --- | --- |
| 502 | Can you tell me about what happened to you after you had been referred? | *Open question serving to get an impression of the patient´s pathway.* | |
| **Referral Hospital 1** (Please fill for **first referral**. If not referred, continue 701) | | | |
| **Treatment 1a** | | | |
| 503 | Where were you referred to? | 1. General Hospital 2. Secondary referral hospital 3. St. Paul Millenium Hospital 4. Black Lion Hospital 5. Private clinic 6. Other: 7. Not answered | If “other”, please ask for specification. |
| 504 | Did you have an appointment at the referral facility? | 1. Yes 2. No (continue 701) 3. Not answered |  |
| 505 | What did they do at this referral facility? | 1. Recommend to start treatment/diagnostics at this referral facility? 2. Refer to another facility (continue 518) 3. Not answered | If “other”, please ask for specification. |
| 506 | Which procedure did they recommend you to start at this referral facility? | 1. FNAC 2. Biopsy 3. Imaging (CT, MRI) 4. Other diagnostics: 5. Surgery 6. Hormonal therapy 7. Chemotherapy 8. Radiation 9. Other treatment: 10. Don´t know 11. Not answered | If “other”, please ask for specification. |
| 507 | Did you start treatment/diagnostics at this referral facility? | 1. Yes 2. No 3. Planned (continue 801) 4. Not answered (continue 801) |  |
| 508 | When did you start treatment/diagnostics at the referral facility? |  | *DD/MM/YYYY*  Use calendar method.  If not answered enter 01/01/1888 |
| 509 | Were you recommended to get further treatment/diagnostics at this referral facility? | 1. Yes 2. No (continue 517) 3. Not answered (continue 517) |  |
| **Treatment 1b** | | | |
| 510 | Which treatment/diagnostics were you recommended to receive? | 1. FNAC 2. Biopsy 3. Imaging (CT, MRI) 4. Other diagnostics: 5. Surgery 6. Hormonal therapy 7. Chemotherapy 8. Radiation 9. Other treatment: 10. Don´t know 11. Not answered | If “other”, please ask for specification. |
| 511 | Did you start treatment/diagnostics at the referral facility? | 1. Yes 2. No (continue 513) 3. Planned (continue 801) 4. Not answered (continue 513) |  |
| 512 | When did you start treatment/diagnostics at the referral facility? |  | *DD/MM/YYYY*  Use calendar method.  If not answered enter 01/01/1888 |
| 513 | Were you recommended to get further treatment/diagnostics at the referral facility? | 1. Yes 2. No (continue 517) 3. Not answered (continue 517) |  |
| **Treatment 1c** | | | |
| 514 | Which treatment/diagnostics were you recommended to receive? | 1. FNAC 2. Biopsy 3. Imaging (CT, MRI) 4. Other diagnostics: 5. Surgery 6. Hormonal therapy 7. Chemotherapy 8. Radiation 9. Other treatment: 10. Don´t know 11. Not answered | If “other”, please ask for specification. |
| 515 | Did you start treatment/diagnostics at the referral facility? | 1. Yes 2. No (continue 517) 3. Planned (continue 801) 4. Not answered (continue 517) |  |
| 516 | When did you start treatment/diagnostics at the referral facility? |  | *DD/MM/YYYY*  Use calendar method.  If not answered enter 01/01/1888 |
| 517 | Were you referred for further treatment? | 1. Yes 2. No (continue 701) 3. Not answered (continue 701) |  |

| **Referral 2** (Please fill for **second referral**. If not further referred, continue 701) | | | |
| --- | --- | --- | --- |
| **Treatment 2a** | | | |
| 518 | Where were you referred to? | 1. Hospital *as specified in 103* 2. General Hospital 3. Secondary referral hospital 4. St. Paul Millennium Hospital 5. Black Lion Hospital 6. Private clinic 7. Other: 8. Not answered | If “other”, please ask for specification. |
| 519 | Did you have an appointment at the referral facility? | 1. Yes 2. No (continue 701) 3. Not answered (continue 701) |  |
| 520 | What did they do at the referral facility? | 1. Recommend to start treatment at the referral facility. 2. Refer (continue 529) 3. Not answered |  |
| 521 | Which treatment/diagnostics did they recommend you to start at the referral facility? | 1. FNAC 2. Biopsy 3. Imaging (CT, MRI) 4. Other diagnostics: 5. Surgery 6. Hormonal therapy 7. Chemotherapy 8. Radiation 9. Other treatment: 10. Don´t know 11. Not answered | If “other”, please ask for specification. |
| 522 | Did you start treatment/diagnostics at the referral facility? | 1. Yes 2. No (continue 528) 3. Planned (continue 701) 4. Not answered | Choose “planned” if appointment is made. |
| 523 | When did you start treatment/diagnostics at the referral facility? |  | *DD/MM/YYYY*  Use calendar method.  If not answered enter 01/01/1888 |
| 524 | Were you recommended to get further treatment/diagnostics at the referral facility? | 1. Yes 2. No (continue 528) 3. Not answered |  |
| **Treatment 2b** | | | |
| 525 | Which treatment/diagnostics were you recommended to receive? | 1. FNAC 2. Biopsy 3. Imaging (CT, MRI) 4. Other diagnostics: 5. Surgery 6. Hormonal therapy 7. Chemotherapy 8. Radiation 9. Other treatment: 10. Don´t know 11. Not answered | If “other”, please ask for specification. |
| 526 | Did you start treatment/diagnostics at the referral facility? | 1. Yes 2. No (continue 528) 3. Not answered |  |
| 527 | When did you start treatment/diagnostics at the referral facility |  | *DD/MM/YYYY*  Use calendar method.  If not answered enter 01/01/1888 |
| 528 | Were you referred for further treatment/diagnostics? | 1. Yes 2. No (continue 701) 3. Not answered (continue 701) |  |

| **Referral 3:** (Please fill for **third referral**. If not referred further, continue 701) | | | |
| --- | --- | --- | --- |
| **Treatment 3a:** | | | |
| 529 | Where were you referred to? | 1. Hospital *as specified in 103* 2. General Hospital 3. Secondary referral hospital 4. St. Paul Millennium Hospital 5. Black Lion Hospital 6. Private clinic 7. Other: 8. Not answered | If “other”, please ask for specification. |
| 530 | Did you have an appointment at the referral facility? | 1. Yes 2. No (continue 701) 3. Not answered |  |
| 531 | What did they do at the referral facility? | 1. Recommend starting treatment at the referral facility. 2. Refer (continue 540) 3. Not answered |  |
| 532 | Which treatment/diagnostics did they recommend you to start at the referral facility? | 1. FNAC 2. Biopsy 3. Imaging (CT, MRI) 4. Other diagnostics: 5. Surgery 6. Hormonal therapy 7. Chemotherapy 8. Radiation 9. Other treatment: 10. Don´t know 11. Not answered | If “other”, please ask for specification. |
| 533 | Did you start treatment/diagnostics at the referral facility? | 1. Yes 2. No (continue 701) 3. Planned (continue 701) 4. Not answered (continue 535) | Choose “planned” if appointment is made. |
| 534 | When did you start treatment/diagnostics at the referral facility? |  | *DD/MM/YYYY*  Use calendar method.  If not answered enter 01/01/1888 |
| 535 | Were you recommended to get further treatment/diagnostics at the referral facility? | 1. Yes 2. No (continue 539) 3. Not answered (continue 539) |  |
| **Treatment 3b** | | | |
| 536 | Which treatment/diagnostics were you recommended to receive? | 1. FNAC 2. Biopsy 3. Imaging (CT, MRI) 4. Other diagnostics: 5. Surgery 6. Hormonal therapy 7. Chemotherapy 8. Radiation 9. Other treatment: 10. Don´t know 11. Not answered | If “other” please specify. |
| 537 | Did you start treatment/diagnostics at the referral facility? | 1. Yes 2. No (continue 539) 3. Planned (continue 701) 4. Not answered (continue 539) |  |
| 538 | When did you start treatment/diagnostics at the referral hospital? |  | *DD/MM/YYYY*  Use calendar method.  If not answered enter 01/01/1888 |
| 539 | Were you referred for further treatment? | 1. Yes 2. No (continue 701) 3. Not answered (continue 701) |  |

| **Referral 4:** (Please fill for **fourth referral**. If not referred further, continue 701) | | | |
| --- | --- | --- | --- |
| **Treatment 4a:** | | | |
| 540 | Where were you referred to? | 1. Hospital *as specified in 103* 2. General Hospital 3. Secondary referral hospital 4. St. Paul Millennium Hospital 5. Black Lion Hospital 6. Private clinic 7. Other: 8. Not answered | If “other” please specify. |
| 541 | Did you have an appointment at the referral facility? | 1. Yes 2. No (continue 701) 3. Not answered |  |
| 542 | What did they do at the referral facility? | 1. Recommend starting treatment at the referral facility. 2. Refer (continue 601) 3. Not answered |  |
| 543 | Which treatment/diagnostics did they recommend you to start at the referral facility? | 1. FNAC 2. Biopsy 3. Imaging (CT, MRI) 4. Other diagnostics: 5. Surgery 6. Hormonal therapy 7. Chemotherapy 8. Radiation 9. Other treatment: 10. Don´t know 11. Not answered |  |
| 544 | Did you start treatment/diagnostics at the referral facility? | 1. Yes 2. No (continue 701) 3. Planned (continue 801) 4. Not answered | Choose “planned” if appointment is made. |
| 545 | When did you start treatment/diagnostics at the referral facility? |  | *DD/MM/YYYY*  Use calendar method.  If not answered enter 01/01/1888 |
| 546 | Were you referred for further treatment/diagnostics? | 1. Yes 2. No (continue 701) 3. Not answered |  |

1. **Additional referrals**

| 601 | How many additional referrals did you experience after being referred from the facility as specified in 540 |  | If not answered enter -99. |
| --- | --- | --- | --- |

1. **Follow-Up**

| 701 | Did you attend any follow-up-visits after the end of treatment? | 1. Yes 2. No (continue 801) 3. Not answered (continue 801) |  |
| --- | --- | --- | --- |
| 702 | Where did you attend the follow-up-visits? | 1. General Hospital 2. Secondary referral hospital 3. St. Paul Millenium Hospital 4. Black Lion Hospital 5. Private Clinic 6. Other: 7. Multiple facilities: 8. Not answered | If “other” or “multiple facilities” please specify. |
| 703 | How many follow-up-visits have you been to since the end of therapy? |  | If not answered enter -99. |
| 704 | When was your last follow-up visit? |  | *DD/MM/YYYY*  Use calendar method.  If not answered enter 01/01/1888 |

1. **End**

| 801 | How many times have you seen a traditional healer before coming to the hospital as specified in Q103? |  | | If not answered enter -99. |
| --- | --- | --- | --- | --- |
| 802 | How many times have you seen a traditional healer after coming to the hsopital as specified in Q103? |  | | If not answered enter -99. |
| 803 | Have you tried holy water before coming to the hospital as specified in Q103? | 1. Yes 2. No 3. Not answered | |  |
| 804 | Have you tried holy water after coming to the hospital as specified in Q103? | 1. Yes 2. No 3. Not answered | |  |
|  | | | | |
| 805 | Looking back, who or what helped you and gave you strengh when following the referral advice? | 1. Familiy 2. Friends 3. Church 4. Village community 5. Staff at the health facilities 6. Other: 7. Not answered | | If “other” please specify. |
| 806 | Which challenges did you encounter, when following the referral advice? | 1. Covering the costs of the health care. 2. Being away from my job for the time of seeking further healthcare. 3. Lack of access to transport 4. Lack of access to information 5. Lack of social support. 6. Finding someone to take over my responsibilities at home (e.g. children) while I was at the hospital. 7. Fear of diagnosis and treatment. 8. Feeling of helplessness 9. Stress 10. Other 11. Not answered | | If “other” please specify. |
| These were all the questions I had. I want to thank you very kindly for taking the time to talk to me. | | | | |
| 807 | Do you have any further questions concerning the interview or the research? | 1. Yes 2. No 3. Not answered | Try to answer question, if not able to, refer to contact number. | |
| 808 | Would you like me to give you a contact number of our team, in case you have any more questions about the research or your disease? | 1. Yes 2. No 3. Not answered | Contact name and number:  Abigiya Wondimagegnehu  091 395 5333 | |

1. **Conduction of the Interview**

| 901 | Name of the interviewer |  |  |
| --- | --- | --- | --- |
| 902 | Was the interview completed until the end (Q804)? | 1. Yes (continue 905) 2. No |  |
| 903 | What was the reason for discontinuation of the interview? | 1. Respondent asked to stop interview. 2. Connection was lost (continue 905). 3. Other (continue 905): | If other, please specify. |
| 904 | Why did the respondent ask to stop the interview? |  |  |
| 905 | What impression of the respondent’s overall sentiments regarding the interview do you have? | 1. Very positive 2. Positive 3. Indifferent 4. Negative 5. Very negative 6. Not to be determined |  |
| 906 | How would you describe the respondent’s overall sentiments regarding the interview in your own words? |  | *Emotions, reactions, willingness to talk etc.* |
| 907 | Signature of the interviewer |  |  |
| 908 | Date of the interview |  | (DD/MM/YYYY) |
| 909 | Length of the interview |  | Minutes |
| 910 | Further comments: |  | |
